# The Maize WRKY Transcription Factor ZmWRKY40 Confers Drought Resistance in Transgenic *Arabidopsis*

**DOI:** 10.3390/ijms19092580

**Published:** 2018-08-30

**Authors:** Chang-Tao Wang, Jing-Na Ru, Yong-Wei Liu, Jun-Feng Yang, Meng Li, Zhao-Shi Xu, Jin-Dong Fu

**Affiliations:** 1 Beijing Advanced Innovation Center for Food Nutrition and Human Health/Beijing Key Lab of Plant Resource Research and Development, Beijing Technology and Business University, Beijing 100048, China; wangct@th.btbu.edu.cn (C.-T.W.); limeng@th.btbu.edu.cn (M.L.); 2 Institute of Crop Science, Chinese Academy of Agricultural Sciences (CAAS)/National Key Facility for Crop Gene Resources and Genetic Improvement, Key Laboratory of Biology and Genetic Improvement of Triticeae Crops, Ministry of Agriculture, Beijing 100081, China; rujingna1993@163.com; 3 Institute of Genetics and Physiology, Hebei Academy of Agriculture and Forestry Sciences/Plant Genetic Engineering Center of Hebei Province, Shijiazhuang 050051, China; liuywmail@126.com; 4 Hebei Wangfeng Seed Industry Co., Ltd., Xingtai 054900, China; Yangjunfenghb@163.com

**Keywords:** WRKY, abiotic stress, regulatory mechanism, drought tolerance, maize

## Abstract

Abiotic stresses restrict the growth and yield of crops. Plants have developed a number of regulatory mechanisms to respond to these stresses. WRKY transcription factors (TFs) are plant-specific transcription factors that play essential roles in multiple plant processes, including abiotic stress response. At present, little information regarding drought-related WRKY genes in maize is available. In this study, we identified a WRKY transcription factor gene from maize, named *ZmWRKY40*. ZmWRKY40 is a member of WRKY group II, localized in the nucleus of mesophyll protoplasts. Several stress-related transcriptional regulatory elements existed in the promoter region of *ZmWRKY40*. *ZmWRKY40* was induced by drought, high salinity, high temperature, and abscisic acid (ABA). *ZmWRKY40* could rapidly respond to drought with peak levels (more than 10-fold) at 1 h after treatment. Overexpression of *ZmWRKY40* improved drought tolerance in transgenic *Arabidopsis* by regulating stress-related genes, and the reactive oxygen species (ROS) content in transgenic lines was reduced by enhancing the activities of peroxide dismutase (POD) and catalase (CAT) under drought stress. According to the results, the present study may provide a candidate gene involved in the drought stress response and a theoretical basis to understand the mechanisms of *ZmWRKY40* in response to abiotic stresses in maize.

## 1. Introduction

Environmental stresses seriously affect plant growth and crop productivity. Meanwhile, traditional crop breeding methods cannot meet the production demands of fine varieties with stress tolerance. Cloning stress-related genes and using genetic engineering techniques to create new crop varieties with higher stress tolerance has become a comparatively effective method. Based on the evidence from previous research, transcription factors (TFs) may be promising potential stress-tolerant candidates [1,2,3,4,5,6]. 

As one of the largest TFs families in plants, WRKYs are characterized by their conservative WRKY domains that can specifically recognize the W-box element in the promoter of its target genes, and play an important role in gene transcription and regulation [7]. Depending on their structural features, the WRKYs can be divided into three basic groups: group I (contains two WRKY domains), group II and III (contain only one WRKY domain) [7]. The zinc finger motif in the WRKY domain (C-X4-5-C-X22-23-H-X1-H or C-X7-C-X23-H-Xe-C, respectively) is different between groups II and III [8]. 

As the first WRKY gene, *SPF1*, was identified in sweet potato [9], many WRKY TFs were identified in different species, including *Arabidopsis* [10], rice [11], soybean [12], and barley [13]. Currently, growing evidence has suggested that WRKY TFs play a central regulatory role in plant response to abiotic stress [14,15]. For instance, the overexpression of *Arabidopsis* WRKY genes *AtWRKY25*, *AtWRKY26*, and *AtWRKY39* enhances heat tolerance, although the expression of *AtWRKY25* and *AtWRKY26* is inhibited by high-temperature stress [16,17]. In addition, the pepper *CaWRKY40* and soybean *GmWRKY13/21/54* genes confer tolerance to different abiotic stresses in transgenic plants [18,19]. Overexpressing the wheat *TaWRKY33* enhanced the drought and heat tolerance of transgenic *Arabidopsis* [20]. WRKYs are involved in various hormone signaling pathways in plants. For example, *AtWRKY40* can recognize the W-box regions of abscisic acid (ABA)-induced genes such as *AtABF4*, *AtABI4*, *AtABI5*, *AtDREB1A*, *AtMYB2*, and *AtRAB18*, and suppress their expression [21]. Exogenous ABA can induce the expression of the cucumber *CsWRKY46* gene, and overexpression of *CsWRKY46* can improve the cold resistance in transgenic plants by regulating related genes in the ABA signaling pathway [22]. These findings confirm that WRKY TFs function as regulators in response to hormones or abiotic stresses.

Because of the wide ecological potential and excellent characteristics of maize (*Zea mays* L.), it is widely cultivated in the temperate zone and in the tropical belt. At present, the output of maize is badly affected by decreased cultivated areas and the ever-worsening environment, especially water scarcity. Even though numerous WRKY genes participate in plant abiotic stress responses, little information is available on the mechanisms of WRKYs in maize. One-hundred and nineteen WRKY genes from the maize B73 genome have been identified, which has made it possible to identify new abiotic stress-related WRKY genes in maize [23]. In this study, the *de novo* transcriptome sequencing of maize (SRP144573) under drought treatment was performed to investigate potential WRKY genes related to maize drought tolerance. We identified a drought-responsive WRKY gene *ZmWRKY40* (GRMZM2G120320), a member of WRKY group II. Overexpression of *ZmWRKY40* promoted root growth and reduced the water loss rates in transgenic *Arabidopsis* under drought stress. This study may provide a foundation to understand the function of WRKY genes in maize drought response.

## 2. Results

### 2.1. De Novo Transcriptome Sequencing Analysis

To find maize stress-responsive genes under drought stress, three-leaf seedlings were dehydrated on filter paper for 4 h, and then were collected for transcriptome sequencing analysis. The results showed that the transcription levels of many genes have been changed after drought treatment (Supplementary Figure S1A). Gene ontology (GO) analyses were used to classify the differentially expressed genes (DEGs) into functional groups, and the DEGs were analyzed against the KEGG database to further understand which pathways the DEGs may be involved in (Supplementary Figure S1B,C). After selecting from the DEGs (the differentially expressed WRKYs are listed in Supplementary Table S2), we chose the gene *ZmWRKY40* (GRMZM2G120320) for the further study.

### 2.2. Phylogenetic Analysis of ZmWRKY40

**Figure 1:**
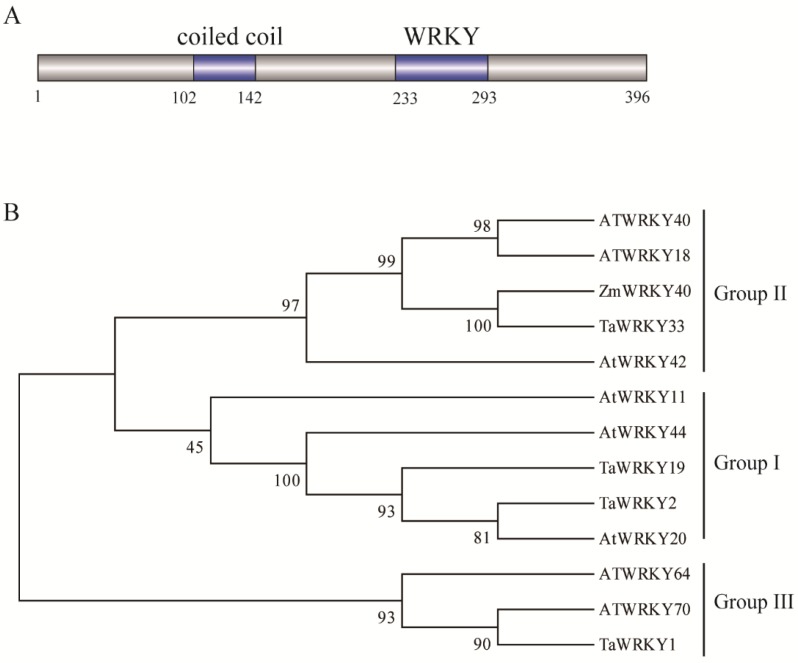
Domain organization and phylogenetic analysis of ZmWRKY40. (**A**) Domain organization of ZmWRKY40. (**B**) Phylogenetic relationship of ZmWRKY40 and other orthologs in different species. Thirteen WRKY proteins from three species were divided into three groups. The phylogenetic tree was produced using the neighbor-joining method with 1000 bootstraps by the MEGA5.0 program.

The complete encoding sequence of *ZmWRKY40* was 1191 bp, encoding 396 amino acids. The ZmWRKY40 protein contained a conserved WRKYGQK domain, a coiled-coil domain (amino acids 102 to 142) at the N-terminus, and a zinc finger motif (C-X5-C-X23-H-X1-H) (Figure 1A). BLASTp online tool was used to search for the homologous amino acid sequences of ZmWRKY40 in wheat and *Arabidopsis*.

According to the criteria of classification of Ulker et al., (2004) [8] and multiple amino acid sequence alignment, five proteins (TaWRKY2, -19; AtWRKY11, -44, and -20) belonged to group I and each contained two WRKY domains; three proteins (AtWRKY64, -70 and TaWRKY1) belonged to group III, five proteins were belonged to group II, and ZmWRKY40 was classified as a member of group II (Figure 1B).

### 2.3. ZmWRKY40 Protein Was Localized in the Nucleus

**Figure 2:**
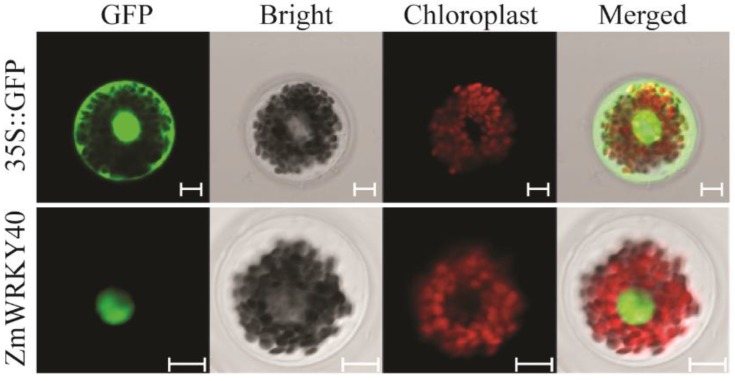
Subcellular localization of ZmWRKY40. ZmWRKY40-green fluorescent protein (GFP) recombinant and p16318hGFP control vector were transiently expressed in maize protoplasts. Scale bars = 10 μm.

To find out the sub-cellular localization of ZmWRKY40 fusion protein, the *ZmWRKY40*-green fluorescent protein (GFP) recombinant was transformed into maize mesophyll protoplasts and the p16318hGFP vector was transformed as a control. As shown in Figure 2, the p16318hGFP protein was expressed in the whole cell, while the ZmWRKY40-GFP fusion protein was localized in the nucleus.

### 2.4. ZmWRKY40 Was Involved in Multiple Abiotic Stresses

Numerous cis-regulatory elements were identified by the online database PLACE (available online: http://www.dna.affrc.go.jp/PLACE/) (Table 1). As shown in Table 1, the promoter region of *ZmWRKY40* contained various abiotic stress-related elements, such as ABA-responsive element (ABRE), MYB, gibberellin (GA)-responsive element (GARE), and W-box, which suggested that *ZmWRKY40* may function in abiotic stress response.

**Figure 3:**
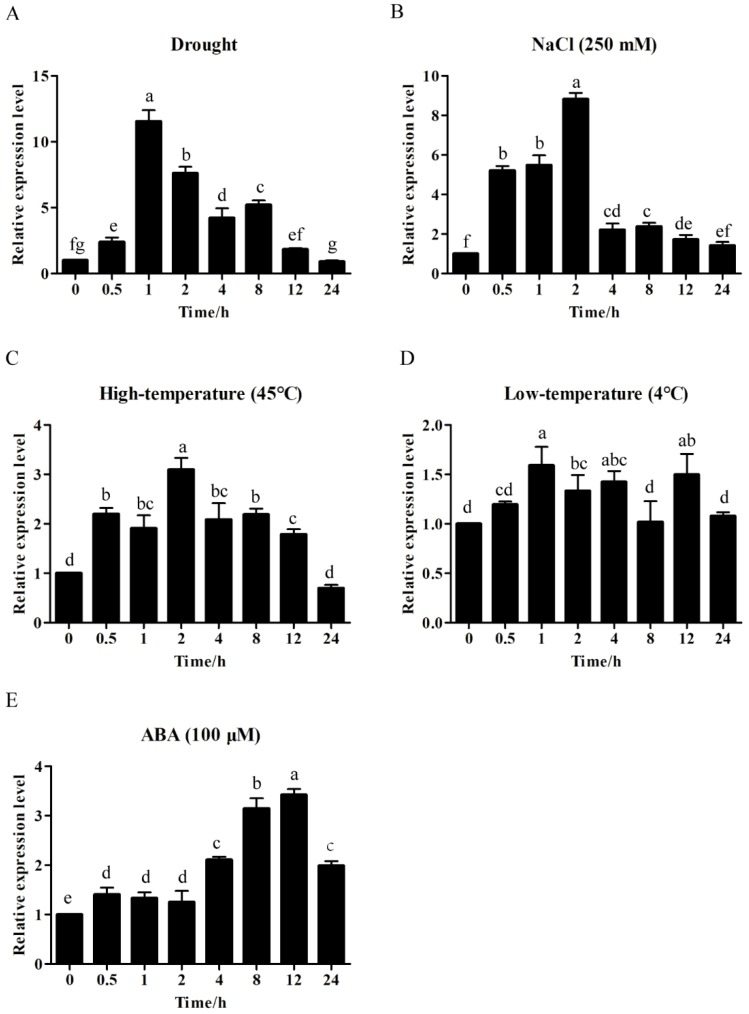
Expression patterns of *ZmWRKY40* under drought (**A**), NaCl (**B**), high-temperature (**C**), low-temperature (**D**), and exogenous ABA (**E**). The vertical ordinates represent fold changes and the horizontal ordinates represent treatment times. Error bar represent standard deviations (SD). The data represent means ± SD of three biological replications. Different letters in bar graphs indicate significant differences at *p* < 0.05. ABA—abscisic acid.

In this study, we investigated the response of *ZmWRKY40* to various abiotic stresses by quantitative real-time PCR (qRT-PCR). The results showed that *ZmWRKY40* was induced by drought, high-temperature, NaCl, and ABA stresses, while it was not affected by low temperature (Figure 3). The expression of *ZmWRKY40* peaked (more than 10-fold) after 1 h of drought treatment. Under salt stress, the transcript of *ZmWRKY40* peaked (8.87-fold) at 2 h, and then declined rapidly to a level similar to the control. Under high-temperature and exogenous ABA treatments, the transcript levels of *ZmWRKY40* were up-regulated and peaked at 2 h (3.08-fold) and 12 h (3.38-fold), respectively.

### 2.5. ZmWRKY40 Enhanced Drought Tolerance in Transgenic Plants

**Figure 4:**
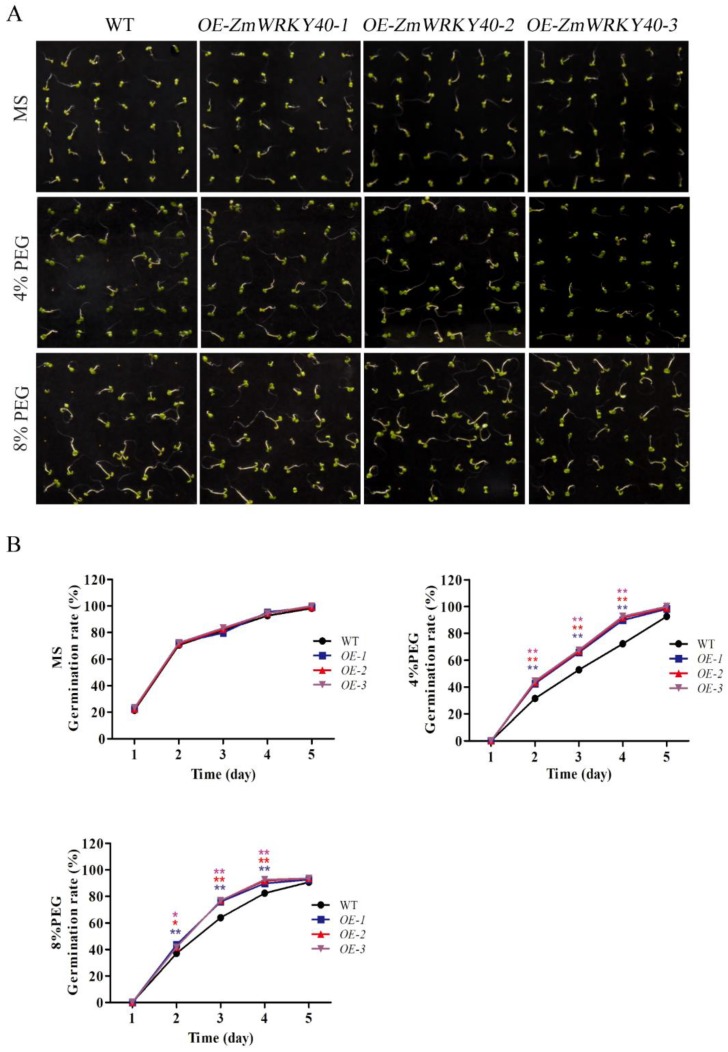
Germination of transgenic *Arabidopsis* lines under drought stress. Seeds were incubated at 4 °C for three days followed by 22 °C for germination. Seeds from three independent transgenic lines with *ZmWRKY40* were grown on MS medium and MS added 4% or 8% PEG6000 (**A**). The germination rate of seeds grown on MS medium and MS medium with 4% or 8% PEG6000 (**B**). All the data represent the means ± SDs of three independent biological replicates and asterisks (**) represent the significant differences at *p* < 0.01 (Student’s *t*-test). WT—wild-type.

**Figure 5:**
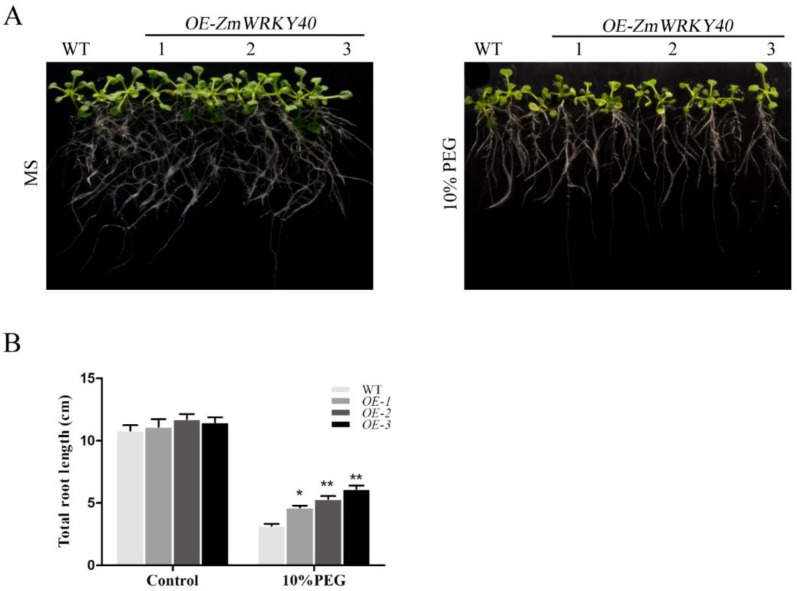
Total root lengths of transgenic *Arabidopsis* lines under drought stress. Five-day-old *Arabidopsis* seedlings were planted on MS medium with or without 10% PEG6000 for 7 days (**A**). The total root lengths were measured (**B**). Data are means ± SD of three independent experiments, and asterisks (* or **) represent the significant differences at *p* < 0.05 or *p* < 0.01, respectively (Student’s *t*-test).

**Figure 6:**
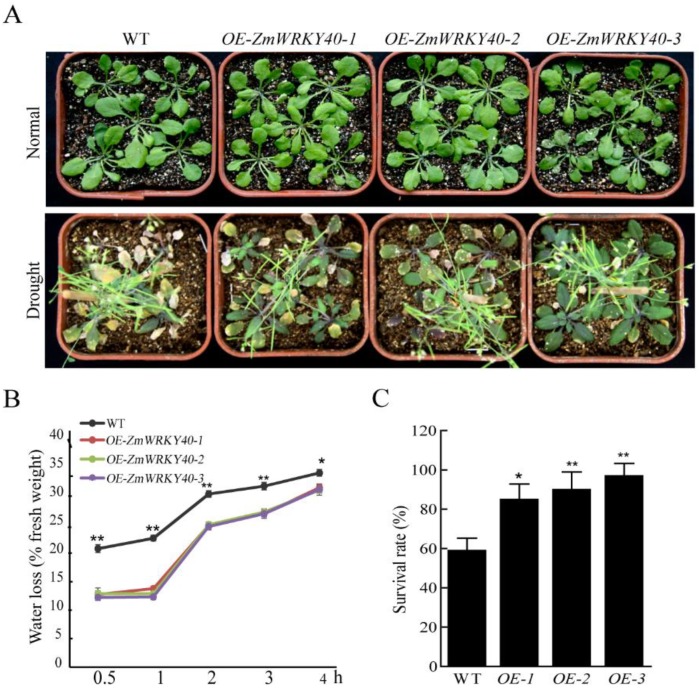
Phenotype analysis and the water loss and survival rates of transgenic *Arabidopsis* and WT under drought treatment. Phenotype analysis of WT and the transgenic *Arabidopsis* under drought treatment (**A**). The water loss rate of WT and transgenic plants under drought condition (**B**). The survival rate of WT and transgenic plants under drought condition was monitored seven days after rewatering (**C**). Data are means ±SD of three independent experiments, and asterisks (* or **) represent the significant differences at *p* < 0.05 or *p* < 0.01, respectively (Student’s *t*-test).

WT was used as the background to transfer the PBI121-*ZmWRKY40*. Three T3 transgenic Arabidopsis lines overexpressing *ZmWRKY40* were selected to be analyzed. Under normal conditions, no differences were observed in seed germination rates between WT and transgenic plants (Figure 4). In the presence of 4% and 8% PEG6000, the germination rate of transgenic seeds was significantly higher than WT (Figure 4). Likewise, the transgenic lines and WT seedlings had little difference in total root length under normal conditions (Figure 5). When exposed to 10% PEG6000, total root lengths of transgenic lines were longer than the WT seedlings after cultivating for seven days, although the growth of both transgenic and WT plants was repressed by PEG6000 (Figure 5). In the seedling stage, there was no significant difference between transgenic and WT plants (Figure 6). However, the transgenic lines were stronger than WT after drought treatment, the survival and water loss rates of transgenic lines and WT plants during drought treatment were measured, and the transgenic lines showed higher survival and lower water loss rates than those of WT plants (Figure 6). Other stress treatments including NaCl treatment in the seedling stage and stomatal apertures experiment under ABA treatment were also carried out, but the *ZmWRKY40*-overexpressing plants exhibited no significant difference under salt and ABA stresses. 

### 2.6. ZmWRKY40 Changes the Expression of Stress-Responsive Gene

**Figure 7:**
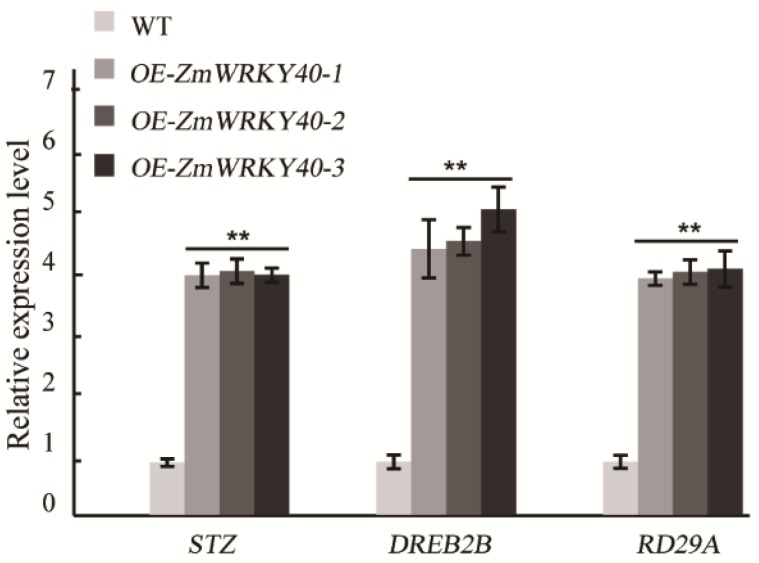
Expression levels of stress-responsive genes in WT and transgenic *Arabidopsis* under normal conditions. The vertical coordinates are fold changes and the horizontal ordinates are gene names. Values are means ± SDs of three replicates, and asterisks (* or **) represent the significant differences at *p* < 0.05 or *p* < 0.01, respectively (Student’s *t*-test).

To further research the possible molecular mechanisms of *ZmWRKY40* in stress responses, the relative expression levels of stress-responsive genes were determined in transgenic and WT plants under normal conditions. The results showed that stress-responsive genes *STZ*, *DREB2B*, and *RD29A* had a more than two-fold increase in transgenic *Arabidopsis* relative to WT plants (Figure 7). These results suggested that *ZmWRKY40* may play a role in drought stress response by regulating stress-related genes.

### 2.7. ZmWRKY40 Changes the Reactive Oxygen Species (ROS) Content and Enzyme Activity

**Figure 8:**
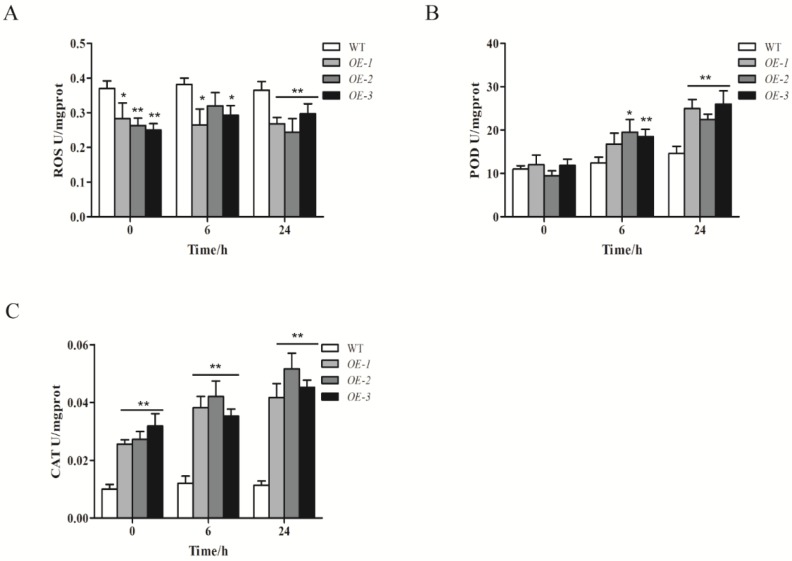
The measurements of physiological-biochemical parameters under normal and drought conditions. The reactive oxygen species (ROS) content (**A**) and the activities of peroxide dismutase (POD) (**B**) and catalase (CAT) (**C**) in WT and transgenic lines were measured. Values are means ± SDs of three replicates, and asterisks (* or **) represent the significant differences at *p* < 0.05 or *p* < 0.01, respectively (Student’s *t*-test).

To better understand the function of *ZmWRKY40* under drought treatment, we assessed the activities of POD and CAT and the ROS content in *ZmWRKY40* transgenic and WT plants at 0 h, 6 h, and 24 h after drought treatment (Figure 8). The ROS content of WT remained at approximately 0.37 U for all of the time points, and the content in transgenic lines had a higher accumulation compared with WT, reaching the maximum at 12 h after drought treatment (Figure 8A). The activities of two antioxidant enzymes, POD and CAT, in transgenic lines were significantly higher than WT plants (Figure 8B,C). As shown in Figure 7B, increases of POD activity were observed in both WT and transgenic lines, but the POD activity in transgenic lines remained higher than in WT plants at all time points, reaching the maximum at 24 h after drought treatment. The activity of CAT was almost unchanged in WT plants under drought treatment, while in transgenic lines, the activity of CAT had a significantly higher level than WT plants (Figure 8C). Overexpression of *ZmWRKY40* reduced the ROS content and enhanced the activities of POD and CAT in transgenic lines under drought stress.

## 3. Discussion

As one of the most serious environmental stresses, drought has a severe effect on the quality and yield of crops. Hence, many studies on drought-related genes have been carried out [1,24,25]. WRKY transcription factors are a class of plant-specific transcription factors that have been mostly reported in the regulation of plant defense responses against biotic stress [26,27,28]. For example, 49 *Arabidopsis* WRKY genes were induced by pathogens or salicylic acid [29]. In addition to playing an important role in biotic stress responses, WRKYs also regulate plant responses to abiotic stresses, such as high salt, drought, and high and low temperatures [8,30,31,32,33]. As the first WRKY protein was isolated from sweet potato [9], many WRKYs have been identified, and numerous WRKYs conferring abiotic stress responses have been studied in many plants. For example, *AtWRKY30* could enhance abiotic stress tolerance during early growth stages in *Arabidopsis* by binding to W-boxes in promoters of many stress-regulated genes [34]. Overexpressing *AtWRKY47* increased drought resistance in *Arabidopsis*, and *AtWRKY57* enhanced drought tolerance in both transgenic *Arabidopsis* and rice plants [35,36]. *OsWRKY30* is activated by MAP kinases to confer drought tolerance in rice, and overexpressing *OsWRKY45* improved salt and drought stress tolerance of transgenic *Arabidopsis* [37]. In wheat, 15 wheat cDNAs were isolated, and 8 genes were responsive to low-temperature, high-temperature, NaCl or PEG treatment [38]. Ten WRKY genes were identified from the genome of wheat, of which *TaWRKY10* enhanced drought and salt stress tolerance in tobacco by regulating the osmotic balance, ROS scavenging, and transcription of stress-related genes [32]. Moreover, GmWRKY27 interacted with GmMYB174 to improve salt and drought tolerance in transgenic soybean hairy roots by suppressing the expression of *GmNAC29* [39]. These results all suggest that WRKYs play important roles in responding to abiotic stresses. 

Maize is one of the most important economic crops, but very few WRKY proteins have been studied in maize, especially regarding their roles in abiotic stress [40]. Since the whole-genome sequencing of maize was completed in 2009, more bioinformatics studies have been conducted to identify the gene families of maize [23,41,42]. For example, a total of 136 WRKYs coded by 119 genes were identified from the maize genome and were numbered [23]. Accordingly, we identified a WRKY gene, *ZmWRKY40*, selected from the drought-treated maize de novo transcriptome data in this study. Similar to other WRKY members, ZmWRKY40 contained one conserved WRKY domain (Figure 1), meaning that it may retain a similar function to other WRKY proteins [23]. To our knowledge, few WRKY genes have been studied in maize. *ZmWRKY17* negatively regulated salt stress tolerance and decreased ABA sensitivity through regulating the expression of some ABA- and stress-responsive genes [43]. *ZmWRKY33* was induced by high-salt, dehydration, cold, and ABA treatments, and it enhanced salt stress tolerance in transgenic *Arabidopsis* [40]. Moreover, *ZmWRKY58* improved tolerance to drought and salt stresses in transgenic rice [44]. These results all suggest that maize WRKYs may play an important role in responding to abiotic stresses.

In our study, *ZmWRKY40* was mainly induced by drought, high-temperature, salt, and exogenous ABA treatments (Figure 3), which may be related to the cis-acting elements of its promoter region (Table 1). For instance, the ABRE and MYB recognition sites in the *ZmWRKY40* promoter region may be responsible for various abiotic stresses and may be involved in ABA signaling. Previous studies have revealed that overexpression of WRKYs in plants can enhance tolerance to drought, salt, cold, and heat stress [14,16,19,33], or can only improve plant tolerance to a single abiotic stress, such as drought [35], salt [40], or heat stress [17]. *ZmWRKY40* improved tolerance to drought in transgenic *Arabidopsis* (Figure 4, Figure 5 and Figure 6), and we also observed the phenotype of *ZmWRKY40*-overexpressing plants in the seedling stage under NaCl treatment, but there was no significant difference. ABA is an important phytohormone, and plays a critical role in regulating plant response to abiotic stresses. Previous studies have demonstrated that there existed ABA-dependent and ABA-independent pathways in drought response [45,46]. Under drought conditions, plants accumulated a high level of ABA, which induced stomatal closure to reduce water loss [47]. The stomatal apertures experiment under ABA treatment was also carried out in our study, but there was no significant difference between *ZmWRKY40*-overexpressing plants and wild-type (WT), which revealed that *ZmWRKY40* was involved in drought stress through ABA-independent signal pathway. ROS are important signaling molecules in the regulation of many of biological processes, and many studies have revealed that the capacity of ROS scavenging was associated with plant tolerance to abiotic stresses [48,49,50,51]. Overexpression of *ZmWRKY40* reduced ROS content and enhanced the activities of POD and CAT under drought treatment (Figure 8), which briefly suggested that *ZmWRKY40* might improve the tolerance to drought by regulating ROS scavenging. Furthermore, overexpression of *ZmWRKY40* activated the expression of stress-responsive genes *STZ*, *DREB2B*, and *RD29A* (Figure 7). STZ, as a transcriptional repressor, enhanced the abiotic stress resistance when overexpressed in transgenic *Arabidopsis* or rice [52]. *DREB* is known to regulate the expression of many stress-inducible genes in the ABA-independent pathways. DREB2B was induced by drought, and overexpressing DREB2B resulted in significant drought tolerance in transgenic *Arabidopsis* plants [53]. *RD29A* contains two major cis-acting elements, the ABRE and the cis-acting DRE, both of which were involved in stress-inducible gene expression [54]. Collectively, these findings show that overexpression of *ZmWRKY40* could improve drought tolerance in transgenic plants possibly by regulating ROS scavenging and enhancing the expression levels of stress-responsive genes. Though *ZmWRKY40* could improve the transgenic *Arabidopsis* resistance to drought, its function in maize still needs to be investigated. Despite this, the function of *ZmWRKY40* in transgenic *Arabidopsis* suggests the future direction of research in maize or other crops.

## 4. Materials and Methods

### 4.1. De Novo Transcriptome Sequencing

Three-leaf stage untreated maize seedlings and seedlings dehydrated on filter paper for four hours were collected for RNA-seq analysis. The detailed process of RNA-seq was exhibited as previously described [20]. The transcriptome data are available in NCBI under accession number SRP144573.

### 4.2. Stress Treatments and Sample Collection

Seeds of maize (X178) used in this study were provided by Dr Zhuan-Fang Hao (Institute of Crop Science, Chinese Academy of Agricultural Sciences, Beijing, China). Wild-type (WT) *Arabidopsis* (Columbia-0) was kept by our laboratory. We planted maize seeds in an incubator before transferring them to pots of 10 cm diameter, with 10 seedlings per pot. The soil contained vermiculite and nutrition soil in a ratio of 1:1 (*v*/*v*). The seedlings were grown in a culture room with 60–70% relative humidity, 25 ± 2 °C, and a photoperiod of 16 h light/8 h dark at a light intensity of around 100 μΜ·m^−2^ s^−2^ until the three-leaf stage [55]. For the heat and cold stress treatments, the seedlings were exposed to 45 °C and 4 °C, respectively. For the exogenous ABA and NaCl treatments, the roots of maize seedlings were soaked in 100 µM ABA and 250 mM NaCl solutions, respectively [40]. For the drought stress treatment, the maize seedlings were placed on filter paper in the culture room [41]. Seedling samples were collected at 0, 0.5, 1, 2, 4, 8, 12, and 24 h after different treatments. The un-treated transgenic and WT *Arabidopsis* were collected to analyze the expression of stress-related genes. The collected samples were dropped into liquid nitrogen for 15 min and then stored at −80 °C.

### 4.3. Reverse Transcription PCR (RT-PCR) and Quantitative Real-Time PCR (qRT-PCR)

The total RNAs were extracted from maize tissue with an RNAprep pure Plant Kit (TIANGEN, Beijing, China). The RT-PCR was conducted using an EasyScript One-Step gDNA Removal and cDNA Synthesis SuperMix kit (TransGen Biotech, Beijing, China). The qRT-PCR was performed with SuperReal PreMix Plus (TIANGEN, Beijing, China) on an ABI Prism 7500 system (Applied Biosystems, Foster City, CA, USA), and each qRT-PCR was repeated three times. The specific primers of *ZmWRKY40* are listed in Supplementary Table S1. The data were analyzed according to the earlier description [56].

### 4.4. Gene Isolation and Bioinformatics Analysis

The full-length of *ZmWRKY40* was amplified from maize cDNA. The primers of *ZmWRKY40*-F and *ZmWRKY40*-R were listed in Supplementary Table S1. The PCR products were cloned into the pEASY-T1 vectors (TransGen Biotech, Beijing, China) and sequenced. The protein functional domains of ZmWRKY40 were predicted by the online analysis tool SMART (available online: http://smart.embl.de/). The amino acid sequence of ZmWRKY40 was used to search the other orthologs from online NCBI database (available online: https://www.ncbi.nlm.nih.gov/) using the BLASTP program. Thirteen WRKY proteins from three species were used for the phylogenetic analysis. The multiple sequence alignments were conducted using ClustalW [57], and the phylogenic tree analysis was performed by MEGA5.0 software with the neighbor-joining (NJ) method [58]. We set default values for all the parameters, and the confidence levels were estimated with bootstrap analyses of 1000 replicates. For further analysis of the transcriptional regulation mechanism of *ZmWRKY40*, *cis*-elements in the promoter region of *ZmWRKY40* were predicted as reported by Zhao et al., 2016 [22].

### 4.5. Subcellular Localization

The expression vector p16318hGFP was used for an investigation of subcellular localization. The coding region of *ZmWRKY40* was fused to p16318hGFP vector containing the CaMV35S promoter. The specific primers were listed in Supplementary Table S1. To confirm the location of ZmWRKY40 fusion protein in cells, the *ZmWRKY40*-GFP recombinant was transformed into maize mesophyll protoplasts by the PEG-mediated method as described by He et al., 2016 [20]. Transfected protoplasts were incubated in darkness at 22 °C for more than 18 h. The fluorescence signals were monitored by a confocal laser scanning microscopy (LSM700; CarlZeiss, Oberkochen, Germany).

### 4.6. Generation of Transgenic Arabidopsis and Its Phenotype under Stress Treatment

The full-length of *ZmWRKY40* was constructed to the plant expression vector pBI121, and the recombinant was transformed to wild-type (WT) *Arabidopsis* (Columbia-0) using the *Agrobacterium*-mediated floral dip method. The specific primers are listed in Supplementary Table S1. The transformed seeds were selected on MS medium containing 50 mM Kanamycin to obtain the positive plants. Three T_3_ generation overexpression lines (*OE-ZmWRKY40-1*, *OE-ZmWRKY40-2*, *OE-ZmWRKY40-3*) with higher expression levels of *ZmWRKY40* were selected by qRT-PCR for further analysis. The method to clean *Arabidopsis* seeds was described by Feng et al., 2015 [1].

Germination assay and root growth assay were used to identify the phenotype under drought stress. For germination assay, seeds of WT and transgenic *Arabidopsis* lines were cultured in MS medium and MS added 4% (*w*/*v*) or 8% PEG6000 for a week. All the media were incubated at 4 °C for three days before moving to 22 °C with a photoperiod of 16 h light/8 h dark. Seeds were considered to be germinated when radicles emerged from the seed coats. For root growth assay, five-day-old seedlings were transferred to MS medium without or with 10% PEG6000 for seven days, and total root lengths of *Arabidopsis* lines were measured [59]. All the experiments were repeated three times.

For the drought treatment, 10-day transgenic and WT plants were transferred to pots filled with a 1:1 mixture of rich soil and vermiculite, and the seedlings were grown at 22 °C with a light intensity of around 100 μΜ·m^−2^ s^−2^ (16 h light/8 h dark photoperiod) under 60% humidity conditions. After growing under normal conditions for three weeks, the drought treatment was imposed by withdrawing irrigation. After severe drought stress for two weeks, water was added for recovery; performance was photographed and the survival rate was monitored seven days after rewatering. Rosette leaves of WT and transgenic plants, which were grown under drought conditions for 7 days, were excised, weighed immediately, and incubated on a bench at room temperature with 60% humidity. Losses in fresh weight were monitored at 0.5 h, 1 h, 2 h, 3 h, and 4 h. Water loss is expressed as the percentage of initial fresh weight. All stress assays were performed three times.

### 4.7. Expression Profile of Stress-Related Genes

To elucidate the possible molecular mechanisms of *ZmWRKY40*, the expression levels of *STZ*, *DREB2B*, and *RD29A* were assessed in transgenic and WT plants under normal condition by qRT-PCR. The specific primers are listed in Supplementary Table S1.

### 4.8. Measurements of Physiological-Biochemical Parameters

The reactive oxygen species (ROS) content, and the activities of peroxide dismutase (POD) and catalase (CAT), were assessed in transgenic and WT plants at 0 h, 6 h, and 24 h after drought treatment as previously described [60].

## 5. Conclusions

We identified a putative drought-responsive WRKY gene, *ZmWRKY40*, a member of group II, from the maize genome by *de novo* transcriptome sequencing (SRP144573). ZmWRKY40 protein was localized in the nucleus. *ZmWRKY40* was induced by drought, high-temperature, NaCl, and ABA treatments. Further research revealed that *ZmWRKY40* could improve drought tolerance in transgenic *Arabidopsis*, and overexpression of *ZmWRKY40* reduced ROS content and enhanced the activities of POD and CAT under drought treatment. Furthermore, ZmWRKY40 changed the expression of stress-responsive genes, including *STZ*, *DREB2B*, and *RD29A*. These results may provide a basis to understand the functions of *ZmWRKY40* in drought resistance in maize.

## Figures and Tables

**Table 1:** Putative cis-elements in the *ZmWRKY40* promoter.

Elements	Number	Sequence	Function
ABRE	6	ACGTG/ACGTSSSC/MACGYGB	ABA- and drought-responsive elements
MYB	2	WAACCA/YAACKG/CTAACCA/CNGTTR/AACGG/TAACAAA	ABA- and drought-responsive elements
GARE	2	TAACAAR	GA-responsive element
ATC-motif	1	TGCTATCCG	Light-responsive element
AuxRe-core	1	GGTCCAT	Auxin-responsive element
W-Box	1	TTTGACY/TTGAC/CTGACY/TGACY	SA-responsive element

ABA—abscisic acid; ABRE—ABA-responsive element; GA—gibberellin; SA—salicylic acid; GARE—GA-responsive element.

## Supplementary Materials

Supplementary materials can be found at http://www.mdpi.com/1422-0067/19/9/2580/s1. Supplementary Table S1. Primers used in the paper. Supplementary Table S2. The differentially expressed *ZmWRKYs* screened from maize *de novo* transcriptome sequencing under drought stress. Supplementary Figure S1. *De novo* transcriptome sequencing analysis of maize under drought stress. (A) The cluster analysis of DEGs under drought treatment. (B) The KEGG analysis of the DEGs between control and drought treatment. The left Y-axis indicated the KEGG pathway. The X-axis indicated the Rich factor. A high q-value was represented by blue, and a low q-value was represented by red. (C) The enrichments of Go terms for DEGs between control and drought treatment.

## Abbreviations

ABA	abscisic acid
ABRE	ABA-responsive element
GA	gibberellin
GARE	GA-responsive element
GFP	green fluorescent protein
qRT-PCR	quantitative real-time PCR
RT-PCR	reverse transcription PCR
SA	salicylic acid
TF	transcription factor
WT	wild type
