# Supplementary material for: The Maize WRKY Transcription Factor ZmWRKY40 Confers Drought Resistance in Transgenic Arabidopsis
Source: Int J Mol Sci. 2018 Aug 30;19(9):2580. doi: 10.3390/ijms19092580 (PMC6164628; doi:10.3390/ijms19092580)
Supplement: Supplementary file 1 [file ijms-19-02580-s001.zip › ijms-335404 supplementary/supplementary figure and table caption.docx]

**Supplementary Table S1**. Primers used in the paper.

**Supplementary Table S2**. The differentially expressed *ZmWRKYs* screened from maize *de novo* transcriptome sequencing under drought stress.

**Supplementary Figure S1**. *De novo* transcriptome sequencing analysis of maize under drought stress. (A) The cluster analysis of DEGs under drought treatment. (B) The KEGG analysis of the DEGs between control and drought treatment. The left Y-axis indicated the KEGG pathway. The X-axis indicated the Rich factor. A high q-value was represented by blue, and a low q-value was represented by red. (C) The enrichments of Go terms for DEGs between control and drought treatment.
