# Supplementary material for: The Maize WRKY Transcription Factor ZmWRKY40 Confers Drought Resistance in Transgenic Arabidopsis
Source: Int J Mol Sci. 2018 Aug 30;19(9):2580. doi: 10.3390/ijms19092580 (PMC6164628; doi:10.3390/ijms19092580)
Supplement: Supplementary file 1 [file ijms-19-02580-s001.zip › ijms-335404 supplementary/Supplemntary Table S1.docx]

| **Table S1. Primers used in the paper.** | | |
| --- | --- | --- |
| **Constructions** | **Primer sequences** |  |
| **Primers for qRT-PCR analysis** | |  |
| *Zm-Actin*-F | GCATCCATGAGACCACCTACAAC |  |
| *Zm-Actin*-R | GATGGACCCTCCTATCCAGACAC |  |
| *RT-ZmWRKY40*-F | CTACTTCCGCTGCTCCTTCG |  |
| *RT-ZmWRKY40*-R | TGCTGCTGCTGGTGCTGCT |  |
| *At-Actin*-F | GAAATCACAGCACTTGCACC |  |
| *At-Actin*-R | AAGCCTTTGATCTTGAGAGC |  |
| *AtRD29A*-F | GTCTGCCGTGACGACGAAGTTAC |  |
| *AtRD29A*-R | TCCTTCTTCTCTTCTTCTCCTCCAA |  |
| *AtSTZ*-F | CGAGGCTCTTACATCACCA |  |
| *AtSTZ*-R | TGTCGCAGACGCTACACTT |  |
| *AtDREB2A*-F | ATGGGAAACCTGGGGAGAAAG |  |
| *AtDREB2A*-R | AAACCGTGTGGTGGCCTTC |  |
| **Primers for gene amplification** |  |  |
| *ZmWRKY40*-F | CCCAGTCCCCAGTCCTTATAT |  |
| *ZmWRKY40*-R | CCAATCTCCCAATCCATCC |  |
| **Primers for subcellular localization** |  |  |
| p16318-*ZmWRKY40*-F | TATCTCTAGAGGATCCATGTCCCGA |  |
| p16318-*ZmWRKY40*-R | TGCTCACCATGGATCCGTCCTTGGTCG |  |
| **Primers for transgenic *Arabidopsis*** | |  |
| pBI121-*ZmWRKY40*-F | CTCTAGAGGATCCCCGGGATGTCCCGA |  |
| pBI121-*ZmWRKY40*-R | ACTAGTGGATCCCCCGGGTCAGTCCTTGGT |  |
