# Supplementary figures and images for: The Maize WRKY Transcription Factor ZmWRKY40 Confers Drought Resistance in Transgenic Arabidopsis
Source: Int J Mol Sci. 2018 Aug 30;19(9):2580. doi: 10.3390/ijms19092580 (PMC6164628; doi:10.3390/ijms19092580)

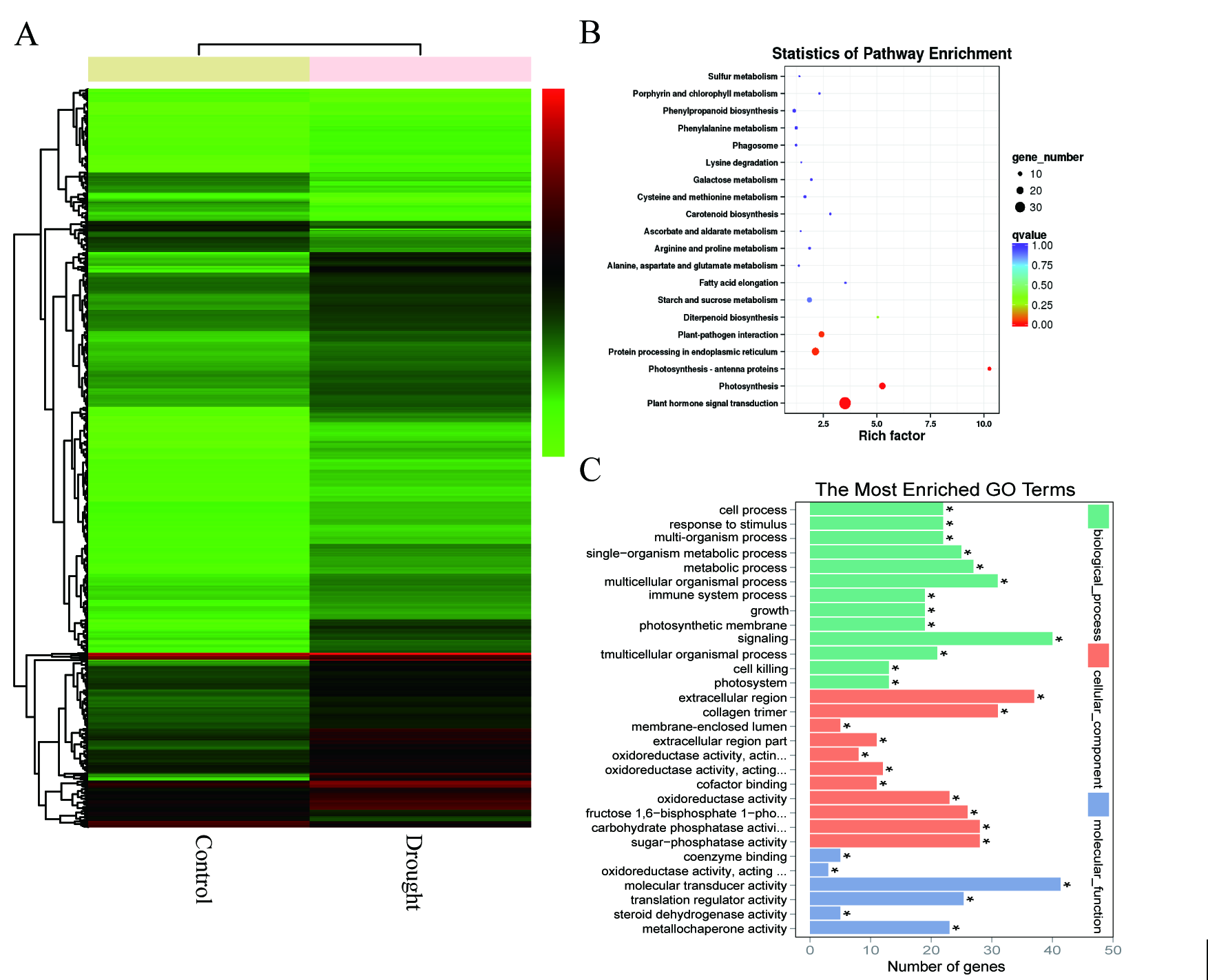

Supplement: Supplementary file 1 [file ijms-19-02580-s001.zip › ijms-335404 supplementary/Figure S1.tif]
